# Causal effects of different types of physical activity on allergic rhinitis: A Mendelian randomization study

**DOI:** 10.1097/MD.0000000000044159

**Published:** 2025-08-29

**Authors:** Xin Yan, Ping Liu, Wei Wang, Junmei Xuan, Mingzhu Shen, Jianghua Peng

**Affiliations:** a Department of Otorhinolaryngology, Shaoxing People’s Hospital (The First Affiliated Hospital, Shaoxing University), Shaoxing, China; b Department of General Practice, Shaoxing People’s Hospital (The First Affiliated Hospital, Shaoxing University), Shaoxing, China.

**Keywords:** allergic rhinitis, C-reactive protein, genetic epidemiology, Mendelian randomization, physical activity

## Abstract

This study utilized Mendelian randomization (MR) with GWAS data to explore causal links between physical activity and allergic rhinitis (AR), aiming to identify lifestyle intervention targets. AR served as the outcome, with exposures including heavy/light do-it-yourself, exercises (e.g., cycling, swimming), and walking-related factors. C-reactive protein (CRP) was set as a mediator, and covariates like vitamin D and air pollution were controlled. Analyses included 2-sample MR, multivariable MR (MVMR), LD score regression (LDSC), and MR mediation. Cycling (IVW: OR = 0.01, *P* = .005) and faster walking pace (OR = 0.47, *P *= .001) were significantly associated with reduced AR risk. LDSC supported genetic correlation for walking pace (*P *= .004). MVMR confirmed independent effects, while MR mediation showed walking pace lowered CRP levels (OR = 0.569, *P *= 2.39e − 15), and elevated CRP increased AR risk (OR = 1.096, *P *= .030), with CRP mediating 6.9% of the protective effect. Cycling and brisk walking may reduce AR risk, with walking’s benefit partially mediated by CRP. These findings highlight physical activity as a potential lifestyle intervention for AR prevention.

## 1. Introduction

Allergic rhinitis (AR), a chronic inflammatory disease of the nasal mucosa mediated by immunoglobulin E (IgE), has a global prevalence ranging from 10% to 40%. With a continuous upward trend, it has emerged as a significant public health concern.^[1,2]^ Its primary symptoms, including nasal itching, sneezing, rhinorrhea, and nasal congestion, severely impact patients’ sleep quality, work efficiency, and mental health. Notably, AR is associated with an increased risk of anxiety and depression.^[3,4]^ The pathogenesis of AR involves complex interactions between genetic susceptibility and environmental factors.^[5,6]^ Established risk factors include air pollutants (such as PM2.5),^[7]^ occupational chemical exposure,^[8]^ smoking,^[9]^ and vitamin D deficiency,^[10]^ and so on. Current treatments mainly rely on antihistamines and intranasal glucocorticoids. However, efficacy remains limited in some patients, and long-term use may cause side effects, underscoring the urgent need to explore novel preventive and interventional strategies.^[11]^

Recent studies suggest that physical activity may influence the risk of AR by modulating the immunoinflammatory response. However, existing evidence is significantly contradictory. For instance, some studies have found that regular exercise can reduce the incidence of AR,^[12,13]^ potentially by suppressing the Th2-type immune response or decreasing the levels of inflammatory markers such as C-reactive protein (CRP).^[14]^ Conversely, other research indicates that high-intensity exercise may exacerbate symptoms due to transient immunosuppression or increased allergen exposure.^[15]^ These discrepancies likely stem from the inherent limitations of observational studies, which struggle to account for confounding factors. Mendelian randomization (MR), by leveraging genetic instrumental variables, effectively circumvents such biases and enables more reliable causal inference. Therefore, there is an urgent need to employ the MR approach to clarify the associations between specific types of physical activity (such as household chores, walking, and cycling) and AR, as well as the underlying mechanisms.

MR emulates randomized controlled trials using genetic instrumental variables, allowing for robust causal inference between exposures and outcomes.^[16]^ Its key advantage lies in avoiding confounding biases and reverse causation that commonly affect traditional observational studies. In this study, MR serves as the primary methodology to systematically evaluate the causal relationships between physical activity patterns, transportation modes, and AR. Additionally, LD score regression (LDSC) is utilized to explore the genetic correlations between exposures and outcomes. LDSC, through the summary statistics of genome-wide SNP association signals, can preliminarily reveal the shared genetic basis between phenotypes.^[17]^ It is essentially a correlation analysis and cannot substitute for the causal inference provided by MR.

## 2. Methods

### 2.1. Study design

**Figure 1.**
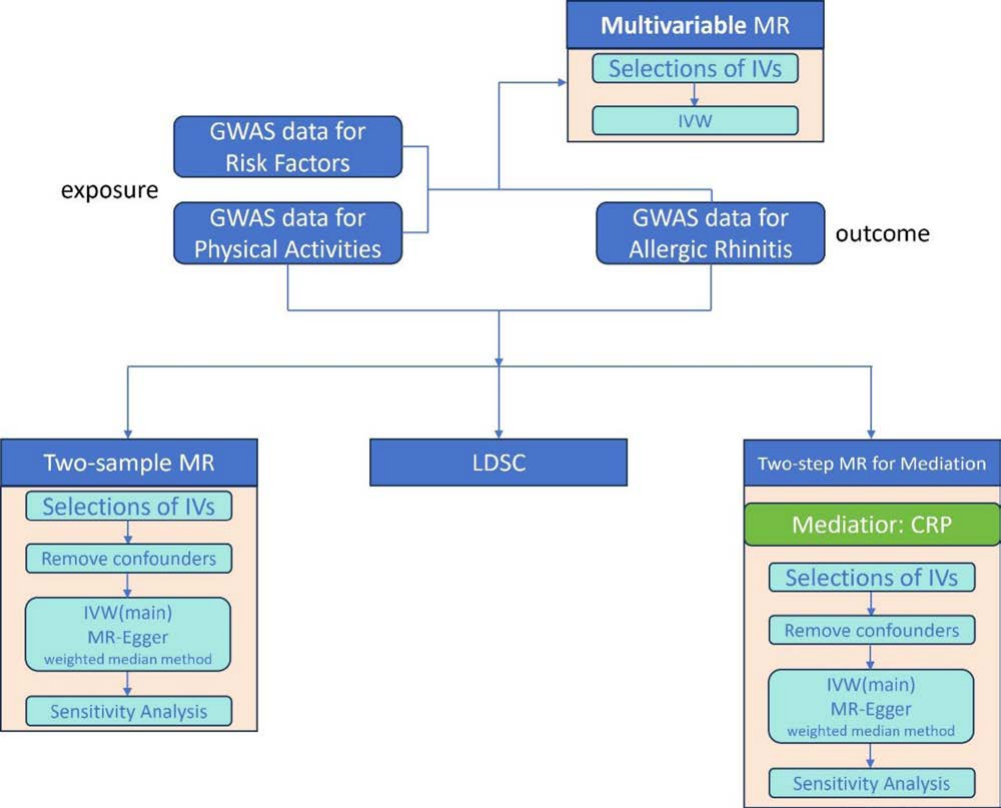
The study design and workflow of the MR study. GWAS = genome-wide association study, IVs = instrumental variables, IVW = inverse variance weighting, LDSC = LD score regression MR = Mendelian randomization.

The study was executed in line with the methodological guidelines of STROBE-MR.^[18]^ To explore the genetic association between physical activity and AR, we employed 2-sample MR to investigate their causal associations, used LDSC to analyze their linear genetic correlation, applied multivariable MR (MVMR) to study the independent causal effects of exposure factors, and used CRP as a mediator to assess mediation effects via a 2-step MR approach. The flowchart of the design is shown in Figure 1.

### 2.2. Data sources and instrumental variable selection

This study utilized publicly available GWAS summary data of European ancestry. Exposure factors included types of physical activity in the last 4 weeks such as heavy do-it-yourself, (4W_Heavy_DIY), light do-it-yourself (4W_Light_DIY), other exercises (4W_Other_Exercises), strenuous sports (4W_Strenuous_Sports), and walking for pleasure (not as a means of transport) (4W_Pleasure_Walk); transportation modes including cycle (excluding work) (Trans_Cycle) and walk (excluding work) (Trans_Walk); as well as walking-related indicators like frequency of walking for pleasure in the last 4 weeks (4W_Pleasure_Walk_Freq), usual walking pace (Walk_Pace), and duration of walks (Walk_Dur). The outcome was AR, with CPR acting as the mediator.

The covariates used for multivariate MR analysis are “Serum 25-Hydroxyvitamin D levels,” “Particulate matter air pollution (pm2.5),” “Workplace full of chemical or other fumes: Often,” “Workplace had a lot of diesel exhaust: Often,” “Cigarettes per Day,” “Frequency of depressed mood in last 2 weeks” (This variable was assessed via a standardized questionnaire in the UK Biobank, with the question: “How often have you felt down, depressed, or hopeless in the past 2 weeks?” Responses included “Never/Rarely/Sometimes/Often/Nearly every day.” The questionnaire items correspond to the validated Patient Health Questionnaire-2 (PHQ-2),^[19]^ reflecting short-term depressive mood.). Details of all GWAS data are shown in Table 1.

**Table 1:** Details of GWAS data.

Role	Trait	Year	Consortium/PMID	Sample size
Exposure	Types of physical activity in last 4 wk: Heavy DIY (e.g.: weeding, lawn mowing, carpentry, digging)	2018	UKB	460,376
Exposure	Types of physical activity in last 4 wk: Light DIY (e.g.: pruning, watering the lawn)	2017	UKB	335,599
Exposure	Types of physical activity in last 4 wk: Other exercises (e.g.: swimming, cycling, keep fit, bowling)	2018	UKB	460,376
Exposure	Types of physical activity in last 4 wk: Strenuous sports	2018	UKB	460,376
Exposure	Types of physical activity in last 4 wk: Walking for pleasure (not as a means of transport)	2018	UKB	460,376
Exposure	Types of transport used (excluding work): Cycle	2018	UKB	460,491
Exposure	Types of transport used (excluding work): Walk	2018	UKB	460,491
Exposure	Duration walking for pleasure	2018	UKB	328,153
Exposure	Frequency of walking for pleasure in last 4 wk	2018	UKB	328,320
Exposure	Usual walking pace	2018	UKB	459,915
Exposure	Duration of walks	2018	UKB	395,831
Mediator	C-reactive protein	2021	34594039	353,466
Outcome	Allergic Rhinitis	2018	FinnGen	490,219
Risk factor	Serum 25-hydroxyvitamin D levels	2020	32242144	496,946
Risk factor	Particulate matter air pollution (2.5 pm); 2010	2018	UKB	423,796
Risk factor	Workplace full of chemical or other fumes: Often	2018	UBK	88,735
Risk factor	Workplace had a lot of diesel exhaust: Often	2018	UKB	89,104
Risk factor	Cigarettes per day	2019	GWAS and sequencing consortium of alcohol and nicotine use	337,334
Risk factor	Frequency of depressed mood in last 2 wk	2018	UKB	442,840

DIY = do-it-yourself, GWAS = genome-wide association study, UKB = UK Biobank.

The criteria for instrumental variable selection were as follows: Select genome-wide significant SNPs (*P *< 5 × 10^−8^, *F* statistic > 10), if the number of SNPs was insufficient, relax the threshold to *P* < 5 × 10^−7^; Perform clustering analysis using PLINK (*r*² < 0.001, window = 10,000 kb) to exclude SNPs with linkage disequilibrium; Remove SNPs that are strongly associated with the outcome (*P* <5 × 10^−8^); In the 2-sample MR and 2-step mediation MR analyses, the SNPs associated with confounding factors (such as body mass index, smoking, etc) were removed simultaneously during the screening of instrumental variables.

### 2.3. Two-sample MR

The inverse variance weighting method (IVW), MR-Egger, and weighted median method were used to assess the causal association between the exposure and AR. If the *P*-value of IVW was <.05 and the effect directions of the 3 methods were consistent, a potential causal association was determined. The false discovery rate (FDR) method was further applied to correct for multiple testing. If the corrected *P*-value was still <.05, a significant causal association was considered to exist.

### 2.4. LDSC

LDSC was used to quantify the genetic correlation between the exposure and AR. A preliminary threshold was set as *P* < .05, and multiple testing was corrected using the FDR method.

### 2.5. MVMR

For the exposure factors that were significant in the 2-sample MR, risk factors for AR (such as PM2.5, occupational chemical exposure, smoking, and vitamin D level) were included for multivariate MR analysis. The IVW model was used to estimate the independent causal effects, and the potential associations between the genetic instrumental variables and covariates were controlled.

### 2.6. Two-step MR for mediation

CRP was chosen as a mediator for its role as a systemic inflammatory marker linked to AR pathogenesis,^[20,21]^ established association with physical activity,^[22,23]^ and availability of high-quality GWAS data (N = 353,466; PMID: 34594039) to support robust mediation analysis. Using 2-sample MR with a 2-step approach, we modeled CRP as the mediating variable: Exposure → CRP: Single nucleotide polymorphisms (SNPs) significantly associated with the exposure (*P* < 5 × 10^−8^) were selected and validated to have no direct association with AR (exclusion restriction assumption). CRP → AR: SNPs strongly associated with CRP (*P* < 5 × 10^−8^) were extracted and confirmed to be independent of the exposure (from Step 1) and the outcome. The mediation effect was estimated by the product method (β_exposure→CRP × β_CRP→AR), and the direct effect was calculated as β_exposure→AR – β_exposure→CRP × β_CRP→AR.

## 3. Sensitivity analysis

To ensure the robustness and reliability of the study results, 3 sensitivity analyses were performed:

1. Assessment of horizontal pleiotropy: The MR-Egger intercept test was employed to detect horizontal pleiotropy. If the *P*-value of the test was <0.05, it indicated that there was bias due to horizontal pleiotropy, violating the MR exclusion restriction assumption.
2. Heterogeneity test: Cochran Q test (based on IVW method) was applied to evaluate heterogeneity among instrumental variables. A significant result (***P* < .05**) suggested substantial heterogeneity, potentially arising from confounding factors, study design variations, or unaccounted genetic correlations.
3. Leave-one-out method verification: The leave-one-out sensitivity analysis was performed, where each SNP was removed one by one, and the causal effect was re-estimated to assess the impact of each SNP on the overall analysis results. If the effect estimate changed significantly after removing a certain SNP, it indicated that this SNP had a crucial impact on the robustness of the results.

### 3.1. Statistical analysis tools

All analyses were conducted in R version 4.3.2, leveraging packages including “2 Sample MR,” “Mendelian Randomization,” and “LDSC” for MR and genetic correlation analyses.

## 4. Results

### 4.1. Two-sample Mendelian randomization

**Figure 2.**
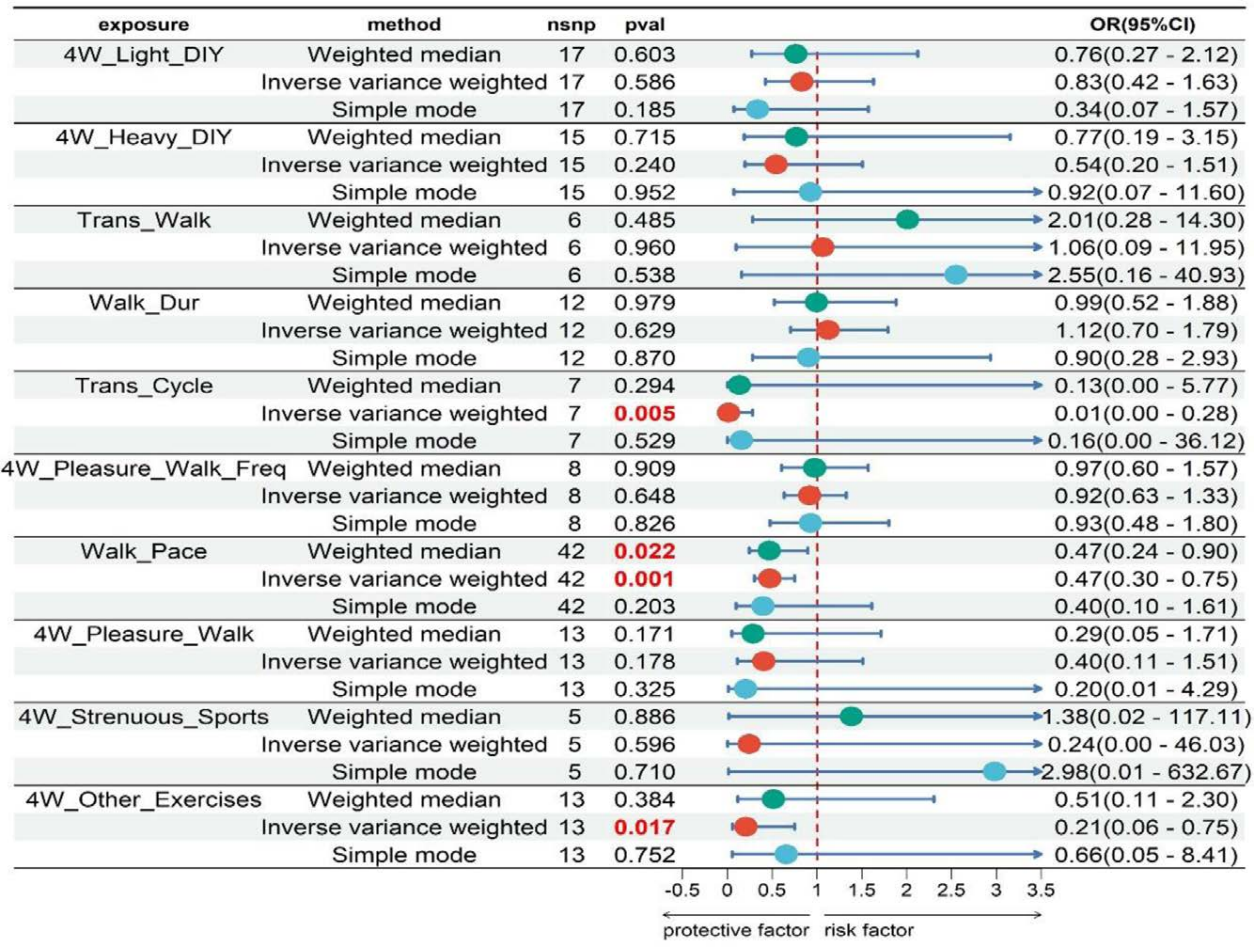
Forest plot of 2-sample MR. 4W_Light_DIY: types of physical activity in last 4 wk: Light DIY; 4W_Heavy_DIY: types of physical activity in last 4 weeks: heavy DIY; Trans_Walk: types of transport used (excluding work): Walk; Walk_Dur: Duration of walks; Trans_Cycle: Types of transport used (excluding work): Cycle; 4W_Pleasure_Walk_Freq: Frequency of walking for pleasure in last 4 wk; Walk_Pace: Usual walking pace; 4W_Pleasure_Walk: types of physical activity in last 4 wk: walking for pleasure; 4W_Strenuous_Sports: types of physical activity in last 4 weeks: strenuous sports; 4W_Other_Exercises: types of physical activity in last 4 weeks: other exercises. CI = confidence interval, MR = Mendelian randomization, NSNP = number of single nucleotide polymorphisms. OR = odds ratio.

After FDR correction, Trans_Cycle and Walk_Pace were significantly associated with a reduced risk of AR. For Trans_Cycle, the odds ratio (OR) was 0.01 (95% confidence interval [CI]: 0.00–0.28, *P*_IVW = 0.005, *P*_FDR = 0.027). For Walk_Pace, the OR was 0.47 (95% CI: 0.30–0.75, *P*_IVW = 0.001, *P*_FDR = 0.014). 4W_Other_Exercises were potential protective factors for AR, with an OR of 0.21 (95% CI: 0.06–0.75, *P*_IVW = 0.017, *P*_FDR = 0.055) (Fig. 2).

### 4.2. LDSC

**Figure 3.**
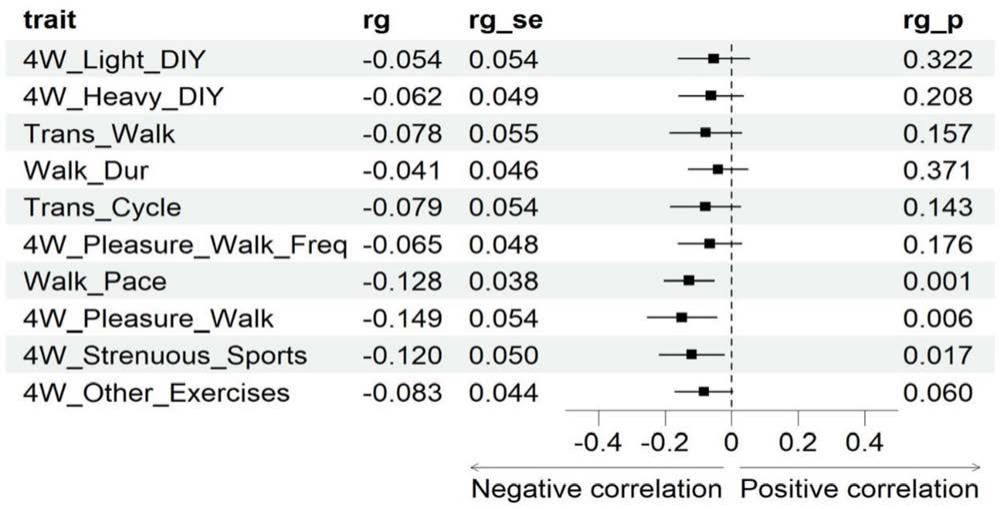
Forest plot of LDSC. 4W_Light_DIY: types of physical activity in last 4 weeks: light DIY; 4W_Heavy_DIY: types of physical activity in last 4 weeks: heavy DIY; Trans_Walk: types of transport used (excluding work): walk; Walk_Dur: Duration of walks; Trans_Cycle: types of transport used (excluding work): cycle; 4W_Pleasure_Walk_Freq: Frequency of walking for pleasure in last 4 wk; Walk_Pace: Usual walking pace; 4W_Pleasure_Walk: Types of physical activity in last 4 weeks: Walking for pleasure; 4W_Strenuous_Sports: Types of physical activity in last 4 weeks: Strenuous sports; 4W_Other_Exercises: Types of physical activity in last 4 weeks: Other exercises; rg: Genetic Correlation Coefficient; rg_se: standard error of genetic correlation coefficient; rg_p: *P*-value of genetic correlation coefficient. LDSC = LD score regression.

Walk_Pace (genetic correlation coefficient[rg] = −0.128, *P* = .001, *P*_FDR = 0.008) and 4W_Pleasure_Walk_Freq (rg = −0.149, *P *= .006, *P*_FDR = 0.028) show a significant negative correlation with AR. 4W_Strenuous_Sports presents a potential negative correlation with AR (rg = −0.120, *P* = .017, P_FDR = 0.055) (Fig. 3).

### 4.3. MVMR

**Figure 4.**
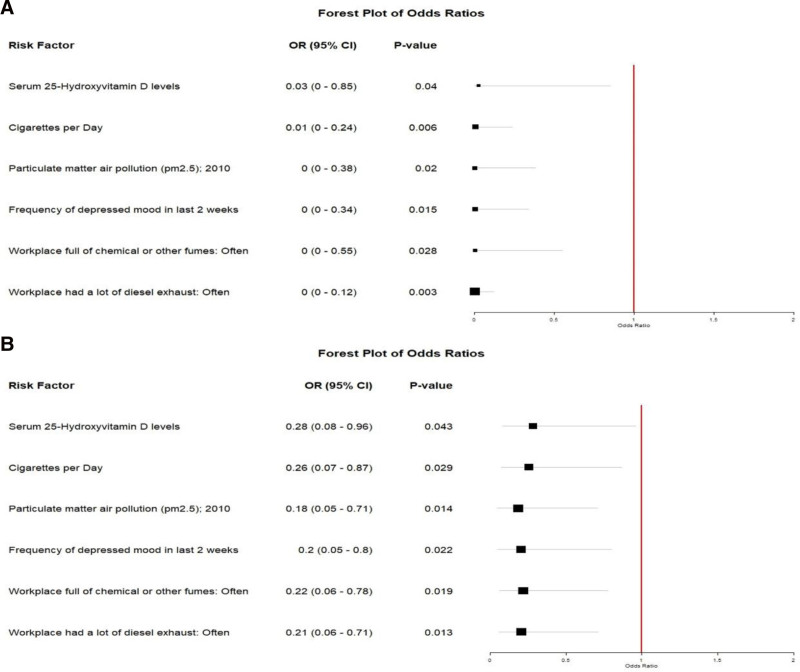
Forest plot of MVMR. (A) represents the effect of Trans_Cycle on AR after sequentially adjusting for risk factors; (B) represents the effect of Walk_Pace on AR after sequentially adjusting for risk factors. AR = allergic rhinitis, CI = confidence interval, MVMR = multivariable Mendelian randomization, OR = odds ratio.

After adjusting for risk factors such as serum 25-hydroxyvitamin D levels, Particulate matter air pollution respectively, the negative effects of Trans_Cycle and Walk_Pace on AR remain significant (Fig. 4).

### 4.4. Two-step MR for mediation

**Figure 5.**
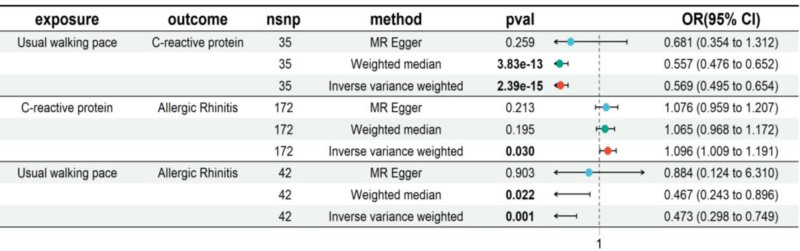
Plot of 2-step MR for mediation. CI = confidence interval, MR = Mendelian randomization, NSNP = number of single nucleotide polymorphisms, OR = odds ratio.

A significant negative causal association was observed between Walk_Pace and CRP (IVW: OR = 0.569, 95% CI = 0.495–0.654, *P* = 2.39 × 10^−15^), while CRP exhibited a positive causal effect on AR (IVW: OR = 1.096, 95% CI = 1.009–1.191, *P* = .030) (Fig. 5). CRP played a partial mediating role in the association between cycling and AR. The mediating effect was −0.052, the direct effect was −0.698, and the proportion of the mediating effect was 0.069.

## 5. Sensitivity analysis

**Figure 6.**
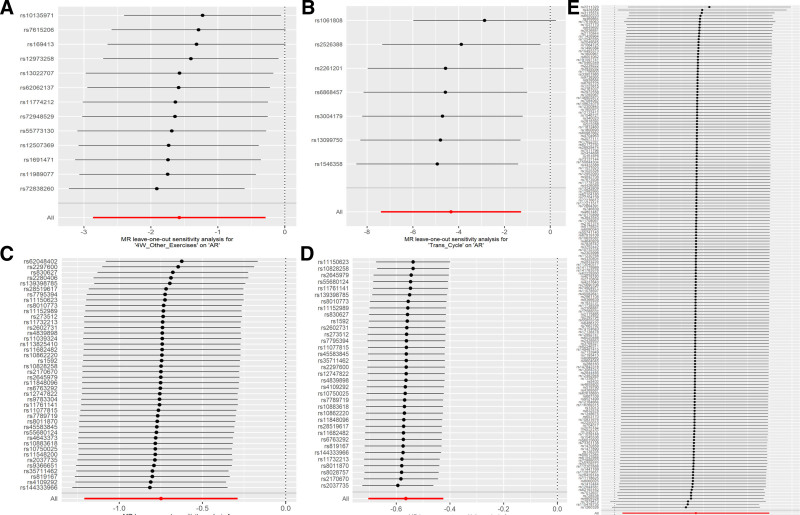
Leave-one-out sensitivity analysis. (A) Sensitivity of the effect of 4W_Other_Exercises on AR; (B) sensitivity of the effect of Trans_Cycle on AR; (C) sensitivity of the effect of Walk_Pace on AR; (D) sensitivity of the effect of Walk_Pace on CRP; (E) sensitivity of the effect of CRP on AR. 4W_Other_Exercises: types of physical activity in last 4 weeks: other exercises: Trans_Cycle: types of transport used (excluding work): cycle. AR = allergic rhinitis, CRP = C-reactive protein, Walk_Pace = usual walking pace.

In the 2-step MR and MR for mediation analyses, sensitivity analyses were conducted. The MR-Egger intercept method indicated that there was no pleiotropy in all MR analyses (*P* > .05). In the Cochran *Q* test, the *P*-values of a small portion of the data were <.05, suggesting that heterogeneity existed in some of the data (Table S1, Supplemental Digital Content, https://links.lww.com/MD/P819). The leave-one-out sensitivity analysis further demonstrated the robustness of the results. The leave-one-out analysis of the MR results with established causal relationships in this study is illustrated in Figure 6.

## 6. Discussion

This study systematically evaluated the causal associations between types of physical activity and AR for the first time using 2-sample MR, LDSC, MVMR, and MR for mediation analysis. The results showed that Trans_Cycle and Walk_Pace significantly reduced the risk of AR (ORs of 0.01 and 0.47, respectively), and CRP mediated 6.9% of the effect. MVMR further indicated that the effect of cycling was independent of confounders such as PM2.5 and smoking. LDSC supported the negative association between cycling and AR (rg = −0.128), consistent with the MR results. Although some exposures showed instrumental variable heterogeneity (possibly due to data sources from different analytical platforms, experimental environments, and study populations), comprehensive sensitivity analyses still validated the robustness of the results, providing high-level causal evidence for AR prevention.

Numerous studies have probed into the complex relationship between physical activity and AR from various aspects. A research on aquatic exercise demonstrated that a 6 – week aquatic training program could augment the sympathetic nerve activity and peak nasal inspiratory flow in young adults with AR, indicating that specific forms of physical activity can optimize nasal respiratory functions.^[24]^ Conversely, investigations focusing on high – intensity exercise among athletes suggested that it might exacerbate AR symptoms, potentially due to transient immunosuppression during strenuous workouts and increased exposure to environmental allergens during outdoor sports.^[25]^ Mechanistically, physical activity influences the autonomic nervous system to alleviate AR symptoms, with postexercise sympathetic nerve activation modulating nasal mucosal blood flow and reducing congestion.^[24]^ Concurrently, exercise induces vasoconstriction in the nasal mucosa, altering the local inflammatory state and improving the nasal mucosal microenvironment as observed in nasal resistance and blood flow studies.^[26]^ Immune-related pathways are central to these effects: physical activity regulates mucosal immune proteins like SPLUNC1, with exercise-induced changes in nasal cavity volume correlating with SPLUNC1 levels in nonsmokers to suggest a role in mucosal barrier function.^[27]^ Physical activity also reduces the release of allergic inflammatory mediators.^[12,28]^ For instance, moderate-intensity winter sports have been shown to alleviate allergic airway inflammation, as evidenced by reduced levels of fractional exhaled nitric oxide and nasal eosinophils.^[12]^

This study reveals differential associations between types of exercise and AR. MR and LDSC analyses highlighted “usual walking pace” as a genetically correlated factor, and MR revealed that cycling, as a transport mode, showed a striking negative association with AR. MVMR analysis showed that the independent protective effects of these 2 exercise modalities are unrelated to confounders such as air pollution and smoking, suggesting that their effects may originate from exercise itself rather than differences in environmental exposure. As moderate-to-low-intensity exercises, both demonstrate significant protective effects against AR, which may be associated with immune balance regulation. Moderate and low-intensity aerobic exercise can favorably modulate the Th1/Th2 ratio and enhance immune system function.^[29]^ Potential protective effects were also observed for other moderate-to-low-intensity exercises such as swimming and fitness. However, the potential negative association with vigorous exercise requires cautious interpretation: high-intensity exercise does not necessarily have beneficial effects on immune responses.^[29]^ While short-term high-intensity exercise may induce elevated stress hormones and transiently suppress mucosal immunity, the bidirectional regulatory effects of long-term regular vigorous exercise on immune function still require verification by more longitudinal studies. This genetically informed approach clarifies causal links, resolving inconsistencies in observational studies by isolating the specific impacts of physical activity intensity and pattern on AR risk.

Exercise may regulate AR by reducing CRP levels, with mechanisms likely involving inhibition of inflammatory pathways and regulation of immune homeostasis. From an inflammatory perspective, CRP, as a classic acute-phase reactive protein, has elevated levels often indicate a chronic inflammatory state in the body. AR is essentially a type I hypersensitivity reaction mediated by IgE, accompanied by eosinophil infiltration in the nasal mucosa and excessive expression of Th2-type cytokines. This study found that regular exercise can reduce systemic inflammatory load by downregulating CRP levels, an effect that may be associated with increased secretion of adipokines (such as adiponectin) induced by exercise,^[22]^ inhibition of monocyte-macrophage activity,^[30]^ or reduction of oxidative stress.^[31]^ For example, in a study on elderly female patients with osteoporosis, 12 weeks of exercise training significantly reduced high-sensitivity CRP (hs-CRP) concentrations, and this change was negatively correlated with osteocalcin concentrations.^[32]^ A comprehensive meta-analysis encompassing 83 randomized and non-randomized controlled trials, involving a total of 3769 participants, demonstrates that exercise training exerts a significant inhibitory effect on CRP levels, with a mean effect size of 0.26. This beneficial impact remains consistent across different age groups and genders. Notably, reductions in body mass index and body fat percentage are independently associated with decreases in CRP levels. These findings collectively validate the role of exercise as a viable intervention strategy for inflammation-related disorders through the modulation of CRP levels.^[23]^

From an AR pathogenesis perspective, previous studies have shown that CRP may be involved in the pathological process of AR, but CRP is not a direct driving factor. However, this does not diminish its value as a marker here. Its sensitivity to systemic inflammatory changes, coupled with its well-established association with physical activity, makes it a valuable indicator with which to explore how immune modulation related to exercise might affect the risk of AR, even if it does not directly cause nasal mucosal inflammation. In a study on children with asthma,^[20]^ elevated CRP was significantly associated with an increased risk of AR complications (the OR increased with rising CRP levels), suggesting that CRP may act as a concomitant marker of inflammatory comorbidities to reflect the overall inflammatory status of the body. However, some studies^[33,34]^ have shown no significant difference in peripheral blood hs-CRP levels between AR patients and healthy controls. This may be related to the local inflammatory nature of AR (predominantly affecting the nasal mucosa) and the limited systemic sensitivity of CRP. An AR immunotherapy study^[21]^ showed that sublingual immunotherapy was found to simultaneously reduce interictal CRP levels and improve rhinitis symptoms. This indirectly suggests that CRP may act as a downstream effector in the inflammatory network, with its level changes being consistent with the clinical efficacy of immunomodulatory therapy. Combined with the MR analysis results of this study, the mediating effect of CRP in the association between “exercise – AR” is weak (mediation proportion is only 6.9%), indicating that the protective effect of exercise against AR may be achieved through multiple pathways. In addition to CRP, this effect may also involve mechanisms such as Th1/Th2 immune balance remodeling, enhancement of nasal mucosal barrier function, or regulation of gut microbiota.^[13,35]^ For example, the analysis of acute-phase proteins in children with AR^[30]^ showed abnormal concentrations of nonspecific immunity-related proteins such as transferrin and α2-macroglobulin in allergic children, suggesting that exercise may exert its effects through broader immunometabolic regulatory pathways, with CRP being only one of the potential mediating nodes.

This study has the following limitations that require cautious interpretation: First, both the genetic instrumental variables and exposure-outcome data are derived from European populations, so extrapolating the conclusions to other ethnic groups must account for heterogeneity in genetic backgrounds and environmental exposures; second, the classification of physical activity types relies on self-reported data, which may weaken classification accuracy due to recall bias or phenotypic heterogeneity; finally, although pleiotropic bias was controlled through sensitivity analyses, instrumental variables may still have potential genetic confounding. Notably, we did not directly adjust for diet, pet exposure, or broader psychological stressors linked to AR. To address confounding, SNPs correlated with these factors were excluded, and MVMR adjusted for vitamin D, PM2.5, occupational chemicals, smoking, and depressed mood. Unmeasured confounding (e.g., unrecorded lifestyle factors) persists, as in observational/MR studies. Nevertheless, based on the robustness of causal inference and the interpretability of biological mechanisms, this study supports cycling and faster walking speed as low-cost intervention strategies for AR. Future research should clarify optimal exercise intervention protocols through multi-ethnic studies and combine objective monitoring with biomarker analysis to reveal individualized mechanisms, thereby promoting the clinical application of precision “exercise prescriptions.”

## 7. Conclusion

This study, using MR analysis, reveals that regular cycling and faster walking pace can significantly reduce the risk of AR, with CRP mediating 6.9% of the effect. MVMR analysis further validates the independence of the cycling effect, suggesting its potential as a preventive strategy for AR. These findings provide genetic causal evidence for the “exercise-inflammatory-allergy relief “ hypothesis and highlight the importance of exercise modality-specific interventions.

## Acknowledgments

We gratefully acknowledge multiple institutions and resources that provided GWAS data for this study, including the UK Biobank for its extensive genetic and phenotypic datasets, the FinnGen project for integrating genomic and health records of the Finnish population, and independent research teams whose published GWAS data supplemented our analyses.

## Author contributions

**Conceptualization:** Xin Yan, Jianghua Peng.

**Data curation:** Ping Liu.

**Formal analysis:** Xin Yan.

**Funding acquisition:** Jianghua Peng.

**Investigation:** Wei Wang.

**Methodology:** Xin Yan.

**Project administration:** Junmei Xuan, Jianghua Peng.

**Resources:** Wei Wang.

**Software:** Ping Liu.

**Supervision:** Junmei Xuan, Jianghua Peng.

**Validation:** Ping Liu.

**Visualization:** Mingzhu Shen.

**Writing – original draft:** Xin Yan.

**Writing – review & editing:** Mingzhu Shen, Jianghua Peng.

## Supplementary Material


