# Supplementary material for: Causal effects of different types of physical activity on allergic rhinitis: A Mendelian randomization study
Source: Medicine (Baltimore). 2025 Aug 29;104(35):e44159. doi: 10.1097/MD.0000000000044159 (PMC12401323; doi:10.1097/MD.0000000000044159)
Supplement: Supplementary file 1 [file medi-104-e44159-s001.docx]

**Supplementary Table 1.** Heterogeneity and pleiotropy analysis in Mendelian randomization

| exposure | outcome | Cochran’s Q_p | Egger_intercept_p |
| --- | --- | --- | --- |
| 4W_Light_DIY | AR | 0.946 | 0.966 |
| 4W_Heavy_DIY | AR | 0.456 | 0.668 |
| Trans_Walk | AR | 0.028 | 0.060 |
| Walk_Dur | AR | 0.533 | 0.175 |
| Trans_Cycle | AR | 0.332 | 0.630 |
| 4W_Pleasure_Walk_Freq | AR | 0.921 | 0.375 |
| Walk_Pace | AR | 0.373 | 0.524 |
| 4W_Pleasure_Walk | AR | 0.331 | 0.282 |
| 4W_Strenuous_Sports | AR | 0.016 | 0.328 |
| 4W_Other_Exercises | AR | 0.055 | 0.473 |
| Walk_Pace | CRP | 1.84e-4 | 0.583 |
| CRP | AR | 3.01e-13 | 0.653 |
